# Ultrasonic Spray Deposition of Hydroxypropyl Methylcellulose Polymer Electrolyte Films

**DOI:** 10.1021/acsomega.6c02740

**Published:** 2026-07-15

**Authors:** Chi-Ping Li, Guan-Xuan Chen

**Affiliations:** Department of Chemical Engineering, 59215 National United University, No. 2, Lienda, Maioli 360302, Taiwan

## Abstract

Gel polymer electrolytes were prepared using an ultrasonic spray deposition (USD) method in this study. The polymer matrix consisted of polyethylene glycol (PEG), with deionized water as the solvent and hydroxypropyl methylcellulose (HPMC) as the thickener. Lithium perchlorate (LiClO_4_) and lithium chloride (LiCl) were used as lithium salts. The effects of spray flow rate, number of coating layers, and lithium salt concentration on transmittance and ionic conductivity were investigated. Surface and cross-sectional morphology were observed using optical microscopy (OM) and scanning electron microscopy (SEM). The results showed that increasing the number of coating layers decreased transmittance due to light absorption by the electrolyte film and lithium salt. The higher lithium salt concentrations, the lower transmittance. Under controllable conditions, the transmittance of the electrolyte with LiClO_4_ exceeded 95%, higher than the typical value reported in the literature (approximately 80%). Ionic conductivity increased with increasing spray flow rate, number of coating layers, and lithium ion concentration. The highest ionic conductivity of the electrolyte with LiClO_4_ reached 3.56 × 10^–5^ S cm^–1^, which compares favorably with previously reported gel polymer electrolytes. These findings indicate that the gel polymer electrolytes developed in this work are qualified for applications in electrochromic device fields.

## Graphical abstract

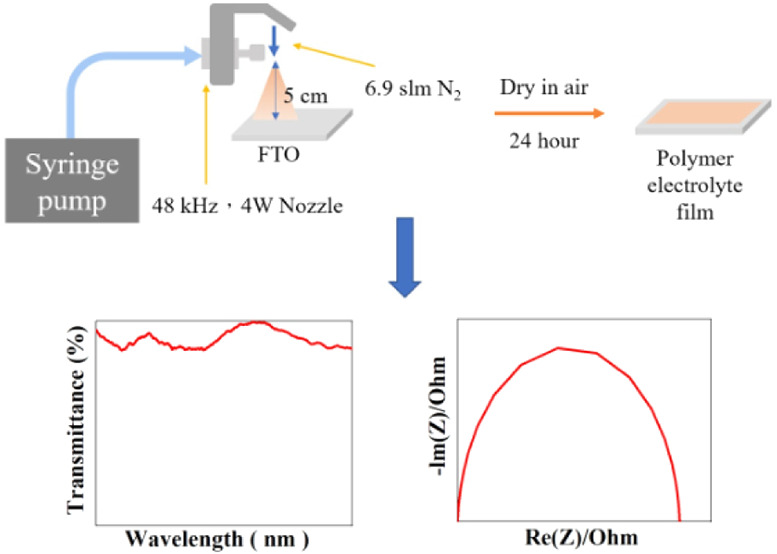

## 1 Introduction

With technological advancements and population growth, global energy demand continues to rise, making sustainable and safe energy storage systems increasingly important, especially in applications such as electric vehicles and smart windows. Lithium-ion batteries play a crucial role due to their high energy density, but traditional liquid electrolytes suffer from problems such as leakage, evaporation, and flammability, posing safety hazards. Solid-state polymer electrolytes (SPEs) and gel polymer electrolytes (GPEs) offer safer and more stable alternatives. This study aims to develop a transparent polymer electrolyte suitable for electrochromic devices and uses an ultrasonic spraying deposition technology for its preparation.

Several polymer materials have been widely used to fabricate solid polymer electrolytes for electrochromic devices. For example, poly­(methyl methacrylate) (PMMA)-based polymer electrolytes have been applied in all-solid-state electrochromic devices with structures such as glass/ITO/WO_3_/electrolyte/ITO/glass. These devices exhibit good optical modulation and stable electrochromic performance, with visible transmittance typically ranging from 80% to 100% depending on operating conditions.^1^ Other solid polymer electrolyte systems include poly­(ethylene oxide) (PEO), poly­(vinylidene fluoride) (PVDF), and poly­(acrylonitrile) (PAN), which can dissolve lithium salts and provide sufficient ionic conductivity for electrochromic switching processes.^2^

Compared with SPEs, gel polymer electrolytes (GPEs) generally exhibit higher ionic conductivity because they contain a liquid electrolyte immobilized within a polymer network. This configuration combines the high ionic conductivity of liquid electrolytes with the mechanical stability of polymer matrices. Typical polymers used to prepare GPEs include poly­(vinyl alcohol) (PVA), poly­(methyl methacrylate) (PMMA), poly­(ethylene oxide) (PEO), and cellulose-derived polymers such as carboxymethyl cellulose (CMC).^3^ These gel systems provide improved electrode–electrolyte contact and faster ion transport, which can significantly enhance electrochromic switching speed and coloration efficiency.

Recent studies have demonstrated several examples of gel polymer electrolytes for electrochromic smart windows. For instance, an interpenetrating polymer network gel electrolyte composed of PVA/CMC/borax has been successfully used in viologen-based electrochromic devices, resulting in fast switching speeds and high optical contrast due to enhanced ionic mobility within the gel matrix.^4^ In another example, PMMA-based gel polymer electrolytes containing plastic crystal additives were reported to improve lithium-ion dissociation and ionic conductivity, thereby enhancing electrochromic device performance.^5^ Furthermore, nanocomposite gel electrolytes incorporating inorganic nanoparticles such as CeO_2_ nanofillers have been shown to improve ion transport pathways and structural stability, leading to electrochromic devices that outperform conventional solution-based electrolytes.^6^

Gel electrolytes have a matrix structure and, like solid electrolytes, do not have leakage problems. Since ions can move freely within the matrix, their ionic conductivity can approach that of liquid electrolytes. Based on these advantages, gel electrolytes have attracted considerable attention for electrochemical energy storage systems and electrochromic devices because of their improved safety, flexibility, and environmental compatibility compared with conventional liquid electrolytes.^7^ Among various polymer systems, polyethylene glycol (PEG) and hydroxypropyl methylcellulose (HPMC) have been widely investigated as promising matrices for gel polymer electrolytes due to their excellent ionic transport capability and mechanical stability. PEG-based polymer electrolytes are known to exhibit efficient ion coordination through ether oxygen groups in the polymer backbone, which facilitates ion migration through the segmental motion of polymer chains. Consequently, PEG-based electrolytes typically exhibit ionic conductivities in the range of 10^–5^–10^–3^ S cm^–1^ at room temperature.^8–10^

HPMC, a cellulose-derived polymer, possesses excellent film-forming capability and strong hydrogen-bonding interactions, enabling the formation of stable three-dimensional gel networks. These networks can effectively retain electrolyte solvents and maintain structural stability during electrochemical operation. Therefore, the incorporation of HPMC into polymer electrolyte systems significantly enhances mechanical strength, electrolyte retention, and long-term stability, which are critical for electrochemical devices such as batteries and supercapacitors.^11–13^

When PEG and HPMC are combined to form composite polymer electrolytes, the resulting polymer blend integrates the high ionic conductivity of PEG with the mechanical robustness of HPMC. The synergistic interaction between PEG and HPMC improves polymer chain mobility and facilitates continuous ion transport pathways within the polymer matrix. As a result, PEG/HPMC composite electrolytes can achieve ionic conductivities approaching 10^–3^ S cm^–1^ at ambient temperature, which is suitable for practical electrochemical energy storage applications.^14–16^

Electrochemical impedance spectroscopy (EIS) analysis of PEG/HPMC polymer electrolytes typically reveals low bulk resistance and efficient ion transport behavior. In addition, linear sweep voltammetry (LSV) measurements demonstrate that polymer gel electrolytes often possess relatively wide electrochemical stability windows, generally ranging from 2.5 to 4.0 V, depending on the polymer composition and electrolyte salts. Such electrochemical stability is sufficient for applications in lithium-ion batteries, supercapacitors, and electrochromic devices.^17–19^

In supercapacitor systems, polymer gel electrolytes based on PEG/HPMC can significantly enhance electrochemical performance by improving electrolyte penetration and ion transport at the electrode–electrolyte interface. Enhanced ionic mobility promotes efficient charge storage through electric double-layer formation and pseudocapacitive reactions. For example, tungsten oxide (WO_3_) and carbon-based composite electrodes paired with polymer electrolytes have demonstrated improved specific capacitance and enhanced electrochemical stability compared with conventional liquid electrolyte systems.^20–22^

Another advantage of PEG/HPMC polymer electrolytes lies in their environmental compatibility and processability. Both PEG and HPMC are water-soluble polymers with relatively low toxicity, allowing for environmentally friendly fabrication processes and compatibility with flexible electronics. In addition, the gel structure provided by HPMC effectively suppresses electrolyte evaporation and improves adhesion between the electrolyte and electrode materials, leading to enhanced cycling stability and long-term performance of electrochemical devices.^23–25^

Recent studies have further demonstrated the potential of PEG/HPMC-based polymer electrolytes for advanced energy storage systems. Mahmood et al. reported that polymer blend gel electrolytes can achieve ionic conductivities as high as 5 × 10^–3^ S cm^–1^ while maintaining excellent electrochemical stability.^26^ Similarly, Ahmed et al. investigated HPMC/PEG–Li_2_SO_4_ polymer blend electrolytes and found that the addition of PEG significantly improves the ion transport properties and thermal stability of the polymer electrolyte matrix.^27^ Chen et al. reported that HPMC-based gel polymer electrolytes can provide fast ion migration channels and high electrochemical stability for sodium-ion battery applications, demonstrating ionic conductivities approaching 10^–3^ S cm^–1^ and wide electrochemical stability windows.^28^ Furthermore, recent reviews on gel polymer electrolytes have highlighted the importance of polymer blending strategies in achieving high ionic conductivity and mechanical durability for flexible energy storage devices.^29^

PEG/HPMC composite polymer electrolytes offer a unique combination of high ionic conductivity, mechanical stability, electrochemical stability, and environmental compatibility. These properties make them promising candidates for next-generation electrochemical systems, including flexible supercapacitors, electrochromic smart windows, and solid-state batteries. Continued research focusing on polymer structure optimization, ion transport mechanisms, and electrode–electrolyte interface engineering is expected to further enhance the performance of PEG/HPMC-based polymer electrolytes.

The ultrasonic spray deposition method can coat large-area surfaces in an environmentally friendly manner while minimizing material waste. The ultrasonic nozzle atomizes the solution according to a predetermined path and can be used to execute a predefined spraying program. In addition, the technology is easy to replicate because the process is relatively simple and can be preprogrammed to produce the desired coating. Compared with other methods, ultrasonic spraying has excellent controllability, efficiency, and scalability, making it a good commercial choice.^30–33^ The samples prepared by ultrasonic spraying exhibit good porosity, uniformity, and distribution of nanoparticles on the surface of the deposited layer.^34–36^ This research uses ultrasonic spray deposition to prepare a PEG/HPMC hybrid gel polymer electrolyte with good transparency and ionic conductivity.

## 2 Experimental Section

### 2.1 Materials

Polyethylene glycol (PEG, Mw = 1000, Aladdin) was used as a plasticizer, hydroxypropyl methylcellulose (HPMC, viscosity 6 mPa·s, Aladdin) was used as the gel polymer matrix. Lithium perchlorate (LiClO_4_, Sigma-Aldrich) and lithium chloride (LiCl, Sigma-Aldrich) were the lithium salts. Deionized water (DIW) was solvent.

### 2.2 Preparation of Polymer Electrolytes

One gram of PEG was dissolved in deionized water, then 0.2 g of HPMC was added, and the mixture was stirred at 65 °C until completely mixed. LiClO_4_ was added to prepare samples with concentrations of 0.4, 0.5, 0.6, and 0.7 M. Similarly, the LiCl concentrations of 0.3, 0.4, and 0.5 M were prepared. Before spraying, the FTO glass (TEC-15, Pilkington, 20 Ω/□) was cleaned in sequence with alcohol, acetone, deionized water, and isopropanol using an ultrasonic vibrator for 3 min, then the FTO was placed in the spraying area below the nozzle. An ultrasonic sprayer (Sonotek) with a frequency of 48 kHz and a power of 4 W was used (Figure 1). The solution was delivered into an injection syringe, and a syringe pump (YSC SP series) was used to send the solution into the ultrasonic spray nozzle. A nitrogen flow with a flow rate of 6.9 slm, as carrier gas, carried the droplets generated by the ultrasonic spray nozzle down to the FTO glass to form polymer electrolyte films. After spraying, another cleaned piece of FTO glass was overlapped with the electrolyte section to form a sandwich structure, and it was left to fully adhere. For the experiment of PEG/HPMC/LiClO_4_ system, samples with flow rates of 0.25 mL min^–1^ with 20 coats and 0.5 mL min^–1^ with 10 and 20 coats were prepared. For the experiment involving the PEG/HPMC/LiCl system, samples with flow rates of 0.5 mL min^–1^ with 10 and 20 coats were prepared.

**Figure 1:**
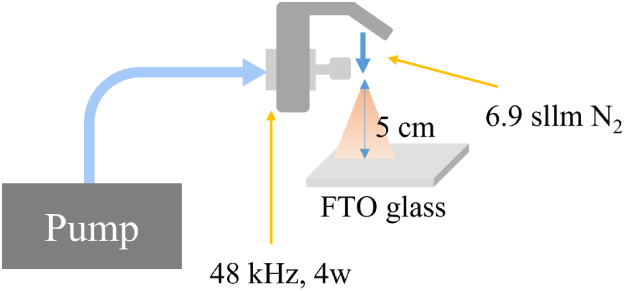
Schematic diagram of the ultrasonic spray system.

### 2.3 Characterization

The optical transmittance of the sample was measured by UV–vis spectroscopy (Bio-Logic SEMSO) at 400–800 nm. Its ionic conductivity was evaluated by electrochemical impedance spectroscopy (EIS) using a Bio-Logic SP-150 potentiostat system. The morphology was observed by optical microscopy (OM, KEYENCE VHX-6000) and scanning electron microscopy (SEM, JSM-5600).

## 3 Results and Discussion

### 3.1 Optical Transmittance

The PEG/HPMC electrolyte films were designed for transparent electrochemical applications. This work systematically evaluates how the composition and deposition conditions affect both ionic transport and optical transparency.

At the same concentration of LiClO_4_, the transmittance decreased as the number of spray layers increased, while the spray flow rate had little effect on the transmittance. When the lithium salt concentration increased, the transmittance did not change accordingly (Figure 2). From Table 1, most of the PEG/HPMC/LiClO_4_ samples demonstrated above 95% transmittance (550 nm), which is excellent as the electrolyte for electrochromic windows.

**Figure 2:**
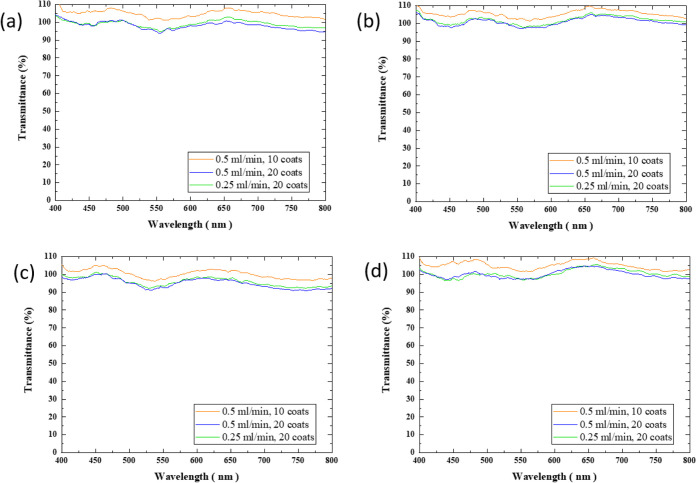
Optical transmittance spectra of different concentrations: (a) 0.4 M, (b) 0.5 M, (c) 0.6 M, (d) 0.7 M of LiClO_4_ at different spraying flow rates and layers.

**Table 1:** Optical Transmittance of PEG/HPMC/LiClO_4_ at Different Lithium Salt Concentrations, Flow Rate, and Spray-Coating Conditions

Lithium salt concentration (M)	Flow rate (mL min^–1^)	Spray coat	Optical transmittance (550 nm) (%)
0.4	0.5	10	99
0.4	0.5	20	94.8
0.4	0.25	20	95.9
0.5	0.5	10	99
0.5	0.5	20	97.8
0.5	0.25	20	98
0.6	0.5	10	97.4
0.6	0.5	20	92.7
0.6	0.25	20	93.6
0.7	0.5	10	99
0.7	0.5	20	97.5
0.7	0.25	20	97.4

When LiCl is at the same concentration, the number of spray layers has little effect on the transmittance, but when the lithium salt concentration was increased, the transmittance decreased. The difference in transmittance can be clearly observed by comparing LiClO_4_ and LiCl at the same concentration and spraying conditions (Figure 3). It can be observed that there were localized agglomerated domains in the LiCl background under an optical microscope (Figure 4). Because LiCl is easy to crystallize, as the LiCl concentration increased, the film surface became rough, which led to an increase in light scattering. Similar phenomena can also be observed in other literature.^37^ In addition, it can be observed that LiCl precipitates as the concentration increased, while LiClO_4_ did not exhibit the same phenomenon (Figure 5). From Table 2, all PEG/HPMC/LiCl samples displayed above 90% transmittance (550 nm), which are also great candidates as electrolytes for electrochromic windows.

**Figure 3:**
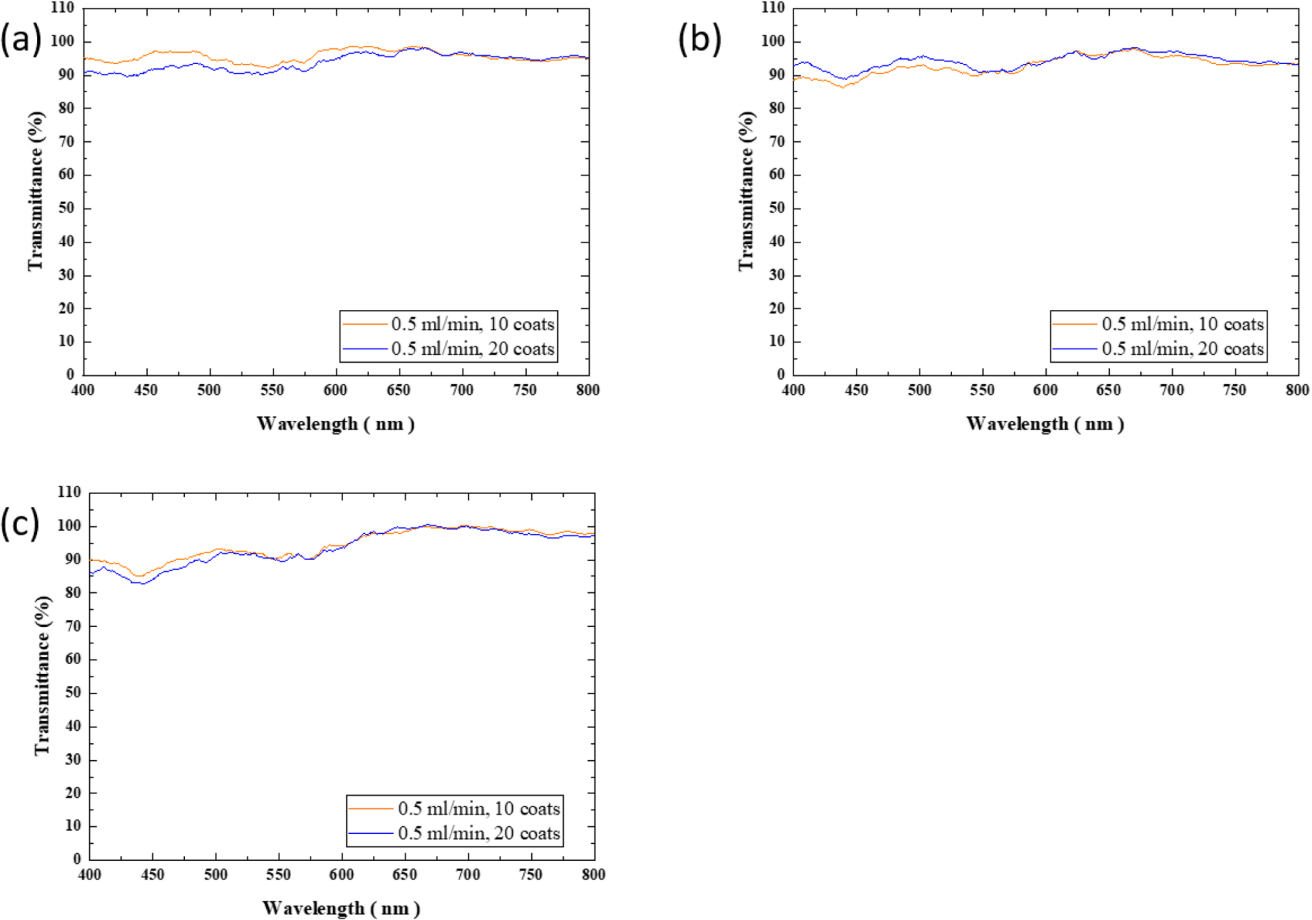
Optical transmittance spectra of different concentrations: (a) 0.3 M, (b) 0.4 M, (c) 0.5 M of LiCl at different spraying flow rates and layers.

**Figure 4:**
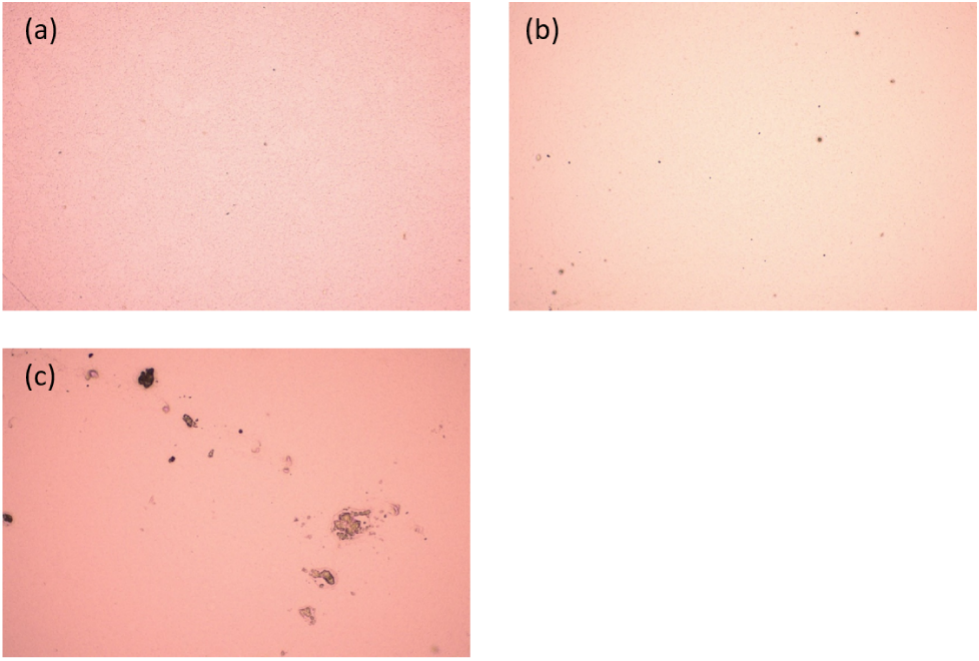
Optical microscopy images of different concentrations of LiCl: (a) 0.3 M, (b) 0.4 M, (c) 0.5 M.

**Figure 5:**
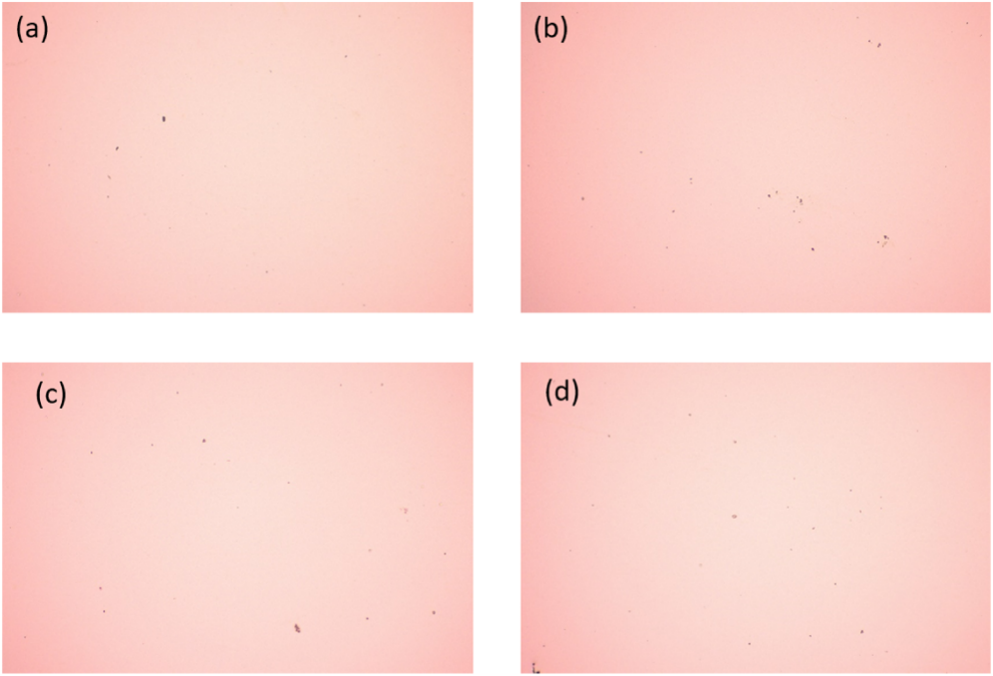
Optical microscopy images of different concentrations of LiClO_4_: (a) 0.4 M, (b) 0.5 M, (c) 0.6 M, (d) 0.7 M.

**Table 2:** Optical Transmittance of PEG/HPMC/LiCl at Different Lithium Salt Concentrations, Flow Rates, and Spray-Coating Conditions

Lithium salt concentration (M)	Flow rate (mL min^–1^)	Spray coat	Optical transmittance (550 nm) (%)
0.3	0.5	10	92.7
0.3	0.5	20	91.0
0.4	0.5	10	90.7
0.4	0.5	20	91.1
0.5	0.5	10	90.5
0.5	0.5	20	89.7

From the SEM cross-sectional images (Figure 6), the film thickness is estimated to be ∼120 μm for samples deposited at 0.25 mL min^–1^ with 20 coating cycles, ∼100 μm at 0.5 mL min^–1^ with 10 cycles, and ∼160 μm at 0.5 mL min^–1^ with 20 cycles. The high optical transparency arises from the uniform polymer matrix and smooth surface morphology (Figures 4 and 5). However, increased film thickness leads to enhanced light scattering within the bulk, thereby causing a slight decrease in optical transmittance.

**Figure 6:**
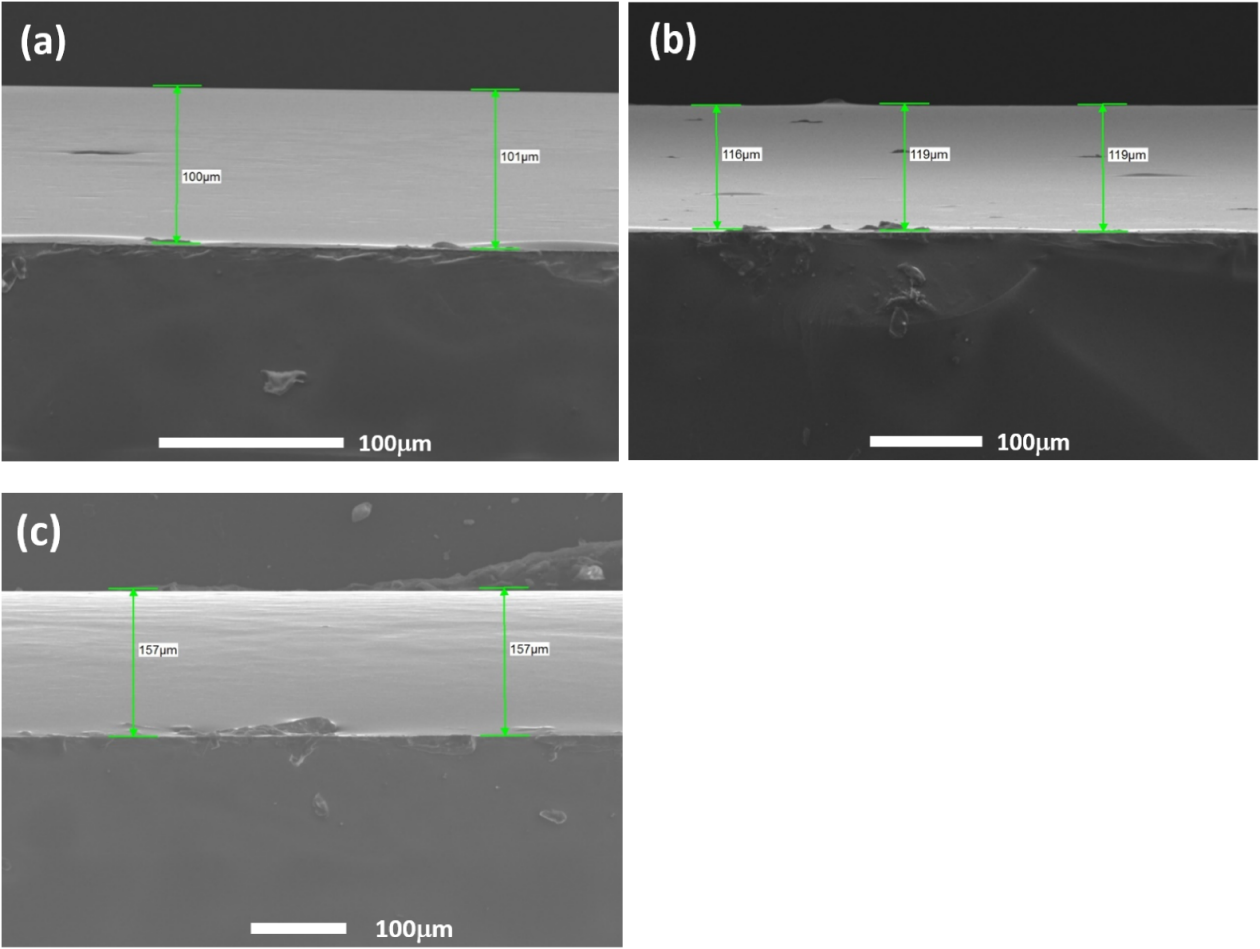
SEM cross-section images of different spray flow rates and spray coats: (a) 0.5 mL min^–1^, 10 coats, (b) 0.25 mL min^–1^, 20 coats, (c) 0.5 mL min^–1^, 20 coats.

It can be seen from Table 3 that, compared with other literature that also uses LiClO_4_ and LiCl as lithium salts, both electrolytes prepared in this study have higher optical transmittance, which is suitable for electrochromic window applications.

**Table 3:** Comparison of Optical Transmittance of Different Materials

Substrate	Sample	Transmittance	Ref.
FTO glass	PEG/HPMC/LiClO_4_	97.5%	This work
FTO glass	PEG/HPMC/LiCl	91.1%	This work
PET-ITO	PEG/LiClO_4_	87.0%	^38^
Conductive glass	LiCl WISE	84.2%	^39^
PET-ITO	PVB/m-PEG//LiClO_4_	83.0%	^40^
ITO glass	PEO/LiClO_4_	75.0%	^41^
FTO glass	PEO/LiCl	63.0%	^42^
FTO glass	EG//LiCl	58.6%	^43^

### 3.2 Ionic Conductivity

As the concentration of LiClO_4_ was increased, the resistance value decreased, indicating that the lithium salt content was increased, the number of mobile ions was increased, and the resistance value decreased (Figure 7).

**Figure 7:**
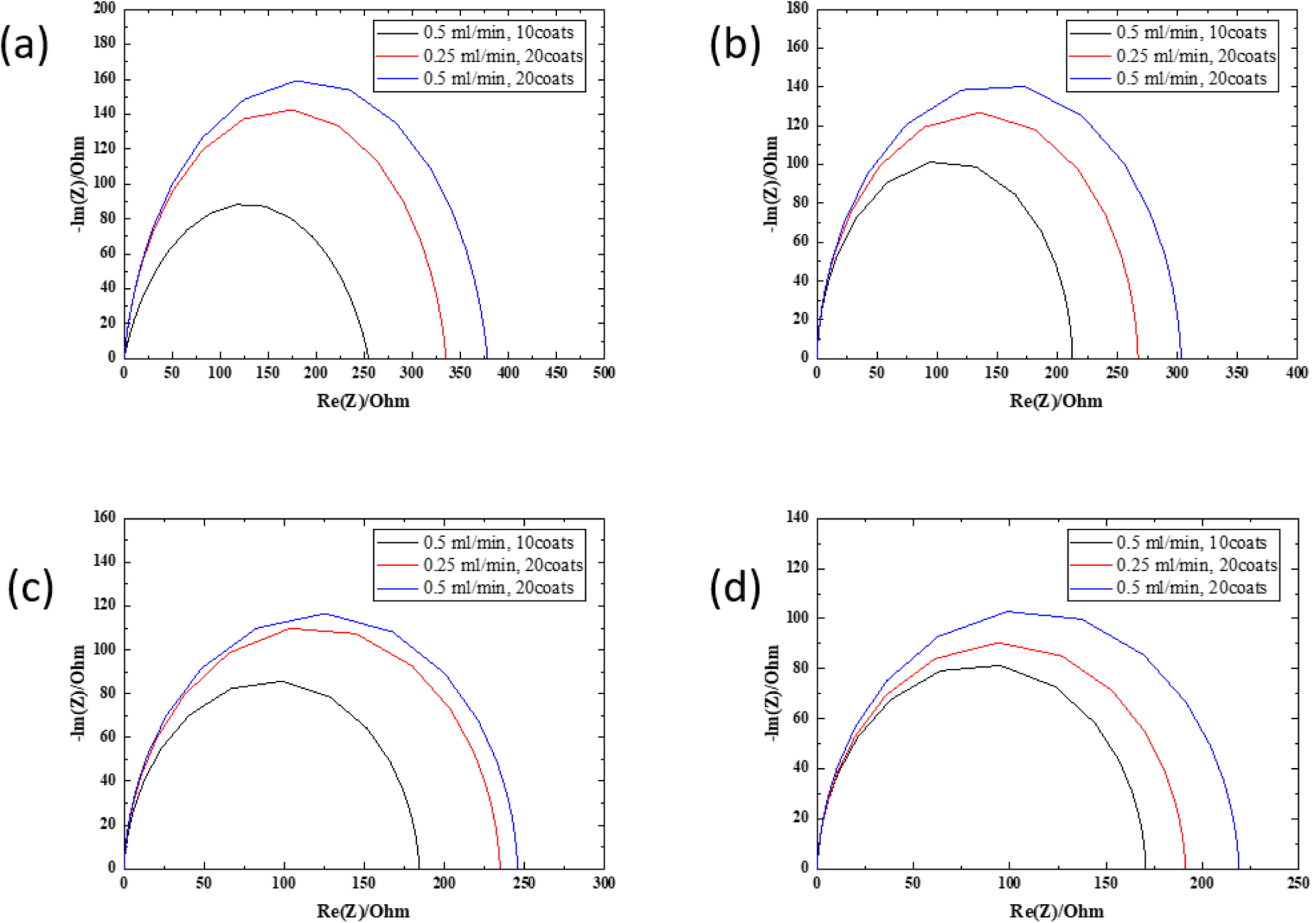
AC impedance diagram of LiClO_4_ at different concentrations: (a) 0.4 M, (b) 0.5 M, (c) 0.6 M, (d) 0.7 M.

As the LiCl concentration was increased, the resistance value first decreased and then increased (Figure 8). The increase is due to the precipitation of the lithium salts. This phenomenon can be confirmed by an optical microscope. As the LiCl concentration increases, the resistance value first decreases and then increases. The increase is due to the precipitation of the lithium salts. This phenomenon can be confirmed by an optical microscope (Figure 4c).

**Figure 8:**
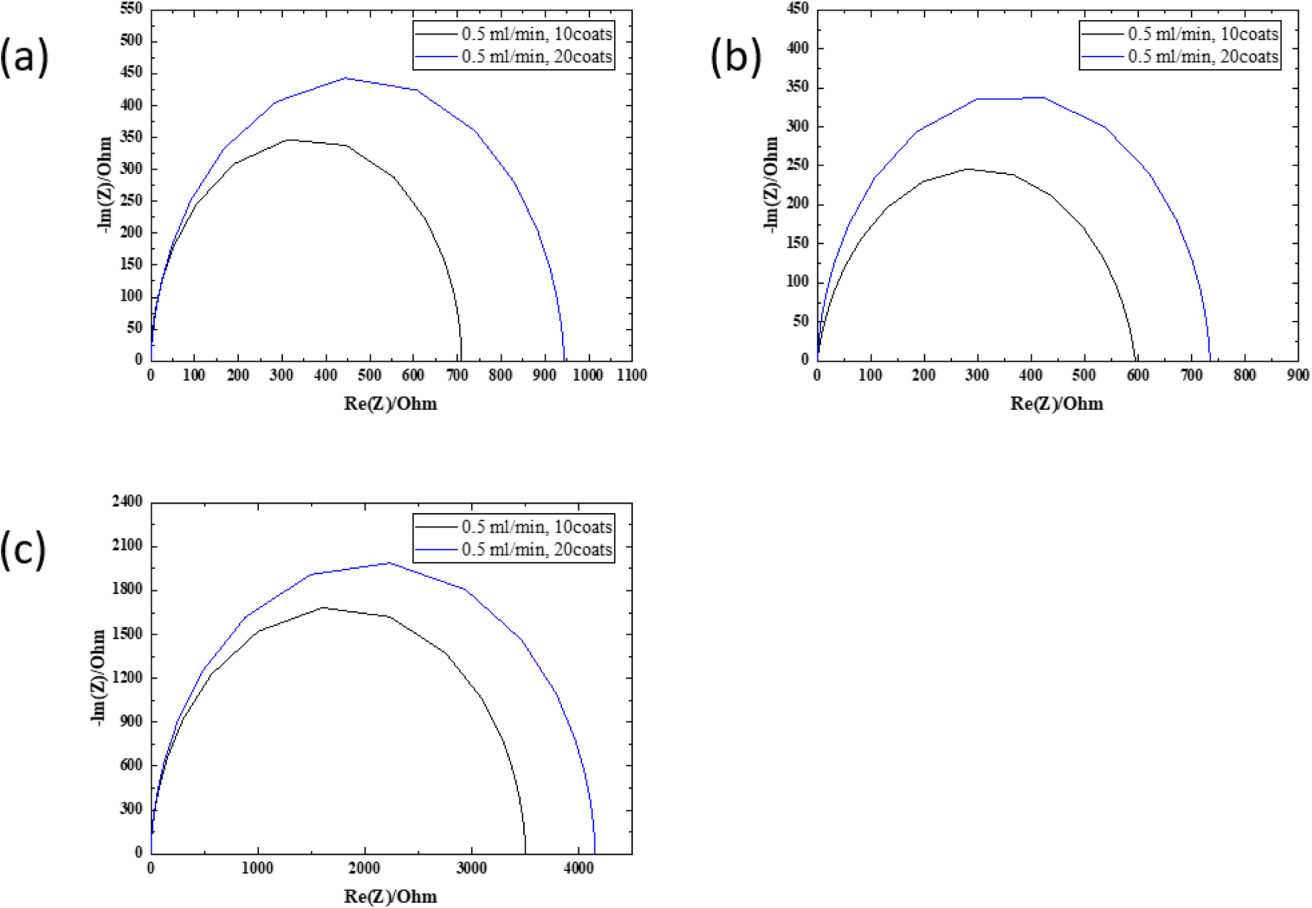
AC impedance diagram of LiCl at different concentrations: (a) 0.3 M, (b) 0.4 M, (c) 0.5 M.

Combined with the film thickness photographed by SEM, the ionic conductivity was calculated as

1 $$\sigma =\frac{L}{A\times R}$$

where *L* is the sample thickness, *R* is the real bulk resistance, and *A* is the area of the sample. From Table 4, the ionic conductivity under the optimal condition—0.7 M LiClO_4_ concentration at a 0.5 mL min^–1^ spray flow rate and 20 layers—is 3.56 × 10^–5^ S cm^–1^. The highest ionic conductivity of LiCl, at a concentration of 0.4 M with a 0.5 mL min^–1^ spray flow rate and 20 coats, is 1.07 × 10^–5^ S cm^–1^ from Table 5.

**Table 4:** Ionic Conductivity of PEG/HPMC/LiClO_4_ at Different Concentrations, Flow Rates, and Spray-Coating Conditions

Concentration (M)	Flow rate (mL min^–1^)	Spray coat	Ionic conductivity (S cm^–1^)
0.4	0.5	10	1.97 × 10^–5^
0.4	0.5	20	2.12 × 10^–5^
0.4	0.25	20	1.78 × 10^–5^
0.5	0.5	10	2.39 × 10^–5^
0.5	0.5	20	2.59 × 10^–5^
0.5	0.25	20	2.23 × 10^–5^
0.6	0.5	10	2.75 × 10^–5^
0.6	0.5	20	3.23 × 10^–5^
0.6	0.25	20	2.56 × 10^–5^
0.7	0.5	10	2.96 × 10^–5^
0.7	0.5	20	3.56 × 10^–5^
0.7	0.25	20	3.13 × 10^–5^

**Table 5:** Ionic Conductivity of PEG/HPMC/LiCl at Different Concentrations, Flow Rates, and Spray-Coating Conditions

Concentration (M)	Flow rate (mL min^–1^)	Spray coat	Ionic conductivity (S cm^–1^)
0.3	0.5	10	7.25 × 10^–6^
0.3	0.5	20	8.34 × 10^–6^
0.4	0.5	10	8.54 × 10^–6^
0.4	0.5	20	1.07 × 10^–5^
0.5	0.5	10	1.44 × 10^–6^
0.5	0.5	20	1.91 × 10^–6^

Compared with other literature where LiClO_4_ and LiCl were also used as lithium salts, it can be found that the electrolyte prepared in this research has superior ionic conductivity, as shown in Table 6. This demonstrates that the polymer electrolyte deposited with LiClO_4_ and LiCl lithium salts in this study is eligible for applications in electrochromic windows. Table 6 compares the ionic conductivity of the PEG/HPMC-based gel polymer electrolytes developed in this work with several polymer electrolyte systems reported in the literature. The highest ionic conductivity obtained in the present study was 3.56 × 10^–5^ S cm^–1^ for the PEG/HPMC/LiClO_4_ electrolyte, while the PEG/HPMC/LiCl electrolyte exhibited a conductivity of 1.07 × 10^–5^ S cm^–1^. Both values are higher than those reported for MMT/LiCl (5.42 × 10^–6^ S cm^–1^),^44^ PEG/Ti­(OEt)_4_/LiCl (4.0 × 10^–6^ S cm^–1^),^45^ and PEG/PVB/LiClO_4_ (2.15 × 10^–6^ S cm^–1^).^46^ The conductivity enhancement observed in the present PEG/HPMC system is likely associated with the hydrophilic nature of PEG and HPMC, which provide abundant ether oxygen and hydroxyl functional groups capable of coordinating with lithium ions and facilitating ion transport through the polymer matrix.

**Table 6:** Comparison of Ionic Conductivity of Different Materials

Sample	Ionic conductivity	Ref.
PEG/HPMC/LiClO_4_	3.56 × 10^–5^ S cm^–1^	This work
PEG/HPMC/LiCl	1.07 × 10^–5^ S cm^–1^	This work
MMT/LiCl	5.42 × 10^–6^ S cm^–1^	^44^
PEG/Ti(OEt)_4_/LiCl	4.0 × 10^–6^ S cm^–1^	^45^
PEG/PVB/LiClO_4_	2.15 × 10^–6^ S cm^–1^	^46^
TPU/PEMPS/LiCl	2.25 × 10^–7^ S cm^–1^	^47^
PTTPAC_2_IL-BF_4_/LiClO_4_	1.10 × 10^–7^ S cm^–1^	^48^
SiO_*x*_C_*y*_H_*z*_/PEG/LiClO_4_	4.60 × 10^–8^ S cm^–1^	^49^

The results also demonstrate that LiClO_4_ produces substantially higher conductivity than LiCl within the same PEG/HPMC matrix. This behavior can be attributed to the larger and more weakly coordinating perchlorate anion (ClO_4_^–^), which promotes greater salt dissociation and increases the concentration of mobile charge carriers. In contrast, the smaller chloride ion exhibits stronger electrostatic interactions with lithium ions, resulting in a lower degree of ion dissociation and, consequently, lower ionic conductivity. Similar trends have been widely reported for PEG- and PEO-based polymer electrolytes.

Compared with TPU/PEMPS/LiCl (2.25 × 10^–7^ S cm^–1^),^47^ PTTPAC_2_IL-BF_4_/LiClO_4_ (1.10 × 10^–7^ S cm^–1^),^48^ and SiOxCyHz/PEG/LiClO_4_ (4.60 × 10^–8^ S cm^–1^),^49^ the conductivities obtained in the present work are approximately one to 3 orders of magnitude higher. This suggests that the PEG/HPMC matrix provides a more favorable environment for ion migration than several previously reported solid polymer electrolyte systems.

Nevertheless, it should be noted that the conductivity values reported here remain lower than those of highly optimized gel polymer electrolytes frequently reported for electrochromic devices and lithium-ion batteries, which often exhibit conductivities in the range of 10^–4^–10^–3^ S cm^–1^. Such systems commonly contain substantial amounts of plasticizers, carbonate solvents, ionic liquids, or nanofillers that enhance polymer segmental mobility and ion dissociation. In contrast, the present PEG/HPMC electrolytes were designed to emphasize film transparency, mechanical integrity, environmentally friendly processing, and compatibility with ultrasonic spray deposition rather than maximizing ionic conductivity. HPMC/PEG-based polymer blend electrolytes containing lithium salts and oxide nanofillers have shown ionic conductivities in the 10^–4^ S cm^–1^ range after optimization. These results indicate that the ionic conductivity of PEG/HPMC/LiClO_4_ polymer electrolytes is strongly influenced by lithium salt dissociation, polymer segmental motion, amorphous phase content, and the presence of plasticizers or inorganic fillers.^50–55^

From an application perspective, the present electrolyte system offers several practical advantages. The PEG/HPMC matrix can be processed by using water-based solutions and deposited uniformly by ultrasonic spray deposition, enabling large-area coating and potential roll-to-roll manufacturing. In addition, the films exhibit acceptable optical transparency and stable film formation, both of which are important characteristics for electrochromic applications. Therefore, although the conductivity is not among the highest reported for gel polymer electrolytes, the present system provides a balanced combination of ionic conductivity, optical transparency, processability, and manufacturing scalability.

The comparison indicates that the PEG/HPMC/LiClO_4_ electrolyte achieves conductivity values superior to those of several previously reported polymer electrolyte systems, while maintaining the advantages of low-temperature processing and scalable film fabrication. Future improvements may be achieved through the incorporation of plasticizers, ionic liquids, or ceramic nanofillers to further increase ion dissociation and polymer chain mobility.

Electrochemical stability is an important factor for polymer electrolytes used in electrochromic devices, as the electrolyte must remain stable within the operating voltage range without decomposition or side reactions. In this study, the primary objective was to demonstrate the feasibility of preparing transparent polymer electrolyte films by ultrasonic spray deposition and to evaluate their optical, ionic conductivity, and mechanical properties. However, electrochemical stability measurements, such as cyclic voltammetry or linear sweep voltammetry, were not included in the present work. This limitation has been acknowledged, and future work will focus on determining the electrochemical stability window of the electrolyte films and further validating their compatibility with electrochromic device operation.

Based on the SEM cross-sectional images (Figure 6), the film thickness varies from ∼100 to 160 μm under different spray conditions. However, the ionic conductivity shows no significant dependence on thickness. This behavior can be explained as follows:

First, the ionic conductivity (σ) is an intrinsic material property, defined by the relationship in eq 1. In this equation, the effect of thickness is mathematically normalized during the conductivity calculation. Therefore, if the internal structure of the film remains similar, σ should be largely independent of thickness.

Second, in the PEG/HPMC polymer electrolyte system, ion transport is primarily governed by the segmental motion of polymer chains. Specifically, polyethylene glycol (PEG) contains flexible ether (−C–O–C−) linkages that coordinate with Li^+^ ions, enabling ion migration through polymer chain dynamics rather than through thickness-dependent pathways. As a result, increasing film thickness does not significantly alter the fundamental ion transport mechanism.

Third, the ultrasonic spray deposition (USD) process produces relatively uniform and continuous films across different coating conditions. SEM observations indicate no significant structural discontinuities, phase separations, or porosity gradients along the thickness direction. This morphological consistency ensures that ion transport pathways remain comparable, regardless of film thickness. This indicates that the observed conductivity is dominated by bulk polymer dynamics rather than by geometric factors.

Compared with conventional solution casting or blade coating, USD enables better control of coating thickness, droplet uniformity, and substrate coverage, while offering a scalable route for large-area manufacturing.

The composite polymer matrix consisted of PEG and HPMC. PEG provides flexible ether chains that facilitate ion transport through segmental motion, whereas HPMC contributes mechanical reinforcement, film-forming ability, and structural stability. The synergistic combination enables simultaneous enhancement of ionic conductivity and film integrity. The PEG phase enhances lithium-ion transport through coordination between Li^+^ ions and ether oxygen groups, while the amorphous character of the polymer matrix promotes segmental mobility. The presence of HPMC helps to retain film integrity while maintaining ion-conduction pathways. HPMC acts as a reinforcing polymer network that improves tensile strength, dimensional stability, and coating adhesion. This is particularly important for thin-sprayed electrolyte layers.

Increasing the spray flow rate increases the amount of precursor delivered to the substrate per unit of time, which can influence film formation. A higher flow rate can promote more complete surface coverage and reduce discontinuities or locally thin regions in the deposited layers. A more continuous electrolyte film provides fewer tortuous ionic transport pathways. The deposition of a larger volume of droplets may slow local evaporation and alter solvent removal behavior during the coating. This can affect the polymer chain packing and salt distribution within the film. Changes in droplet coalescence and drying rate may produce a film morphology that is more favorable for ion migration, such as improved connectivity of amorphous regions. Therefore, the higher conductivity observed at an increased flow rate is attributed to improved film formation and internal transport pathways rather than the flow rate alone.

Increasing the number of spray coats increases the deposited mass and film thickness, but the conductivity trend cannot be explained by thickness alone. Additional coating coats may also lead to the filling of uncovered substrate regions, healing of pinholes or microdefects, improved layer uniformity, more complete electrolyte network formation, and better interparticle/interdomain contact. These structural improvements can reduce the effective resistance and enhance the measured ionic conductivity.

Residual solvents may act as a temporary plasticizing component that enhances polymer segmental motion and facilitates ion transport. Depending on the spray conditions and drying rate, samples prepared at different flow rates or coating thicknesses may retain different amounts of solvent.

The primary objective of the present study was to evaluate the feasibility of USD as a scalable fabrication route for polymer electrolyte films. Future work will include advanced characterization to further verify the proposed mechanism. Although XRD, FTIR, and DSC analyses were not included in this study, indirect evidence of polymer–salt interactions can be inferred from the observed dependence of ionic conductivity and optical transmittance on salt concentration and coating conditions. The systematic variation in conductivity with lithium salt concentration suggests that ion transport is strongly influenced by interactions between the dissolved salt and the polymer matrix, consistent with established behavior reported for PEG- and HPMC-based gel polymer electrolytes in the literature.

Some highly optimized GPE systems reported in the literature achieve conductivities approaching 10^–4^–10^–3^ S cm^–1^. However, these systems often rely on large amounts of liquid plasticizers or carbonate solvents, highly swollen gel structures, ionic liquids, specialized polymer blends, nanofiller-assisted dissociation, and extensively optimized formulations. Such conditions can significantly enhance ion mobility through liquid-like transport pathways. By contrast, the present electrolyte is a spray-coated thin film designed with greater emphasis on process compatibility and dimensional stability. As a result, the conductivity is expected to be lower than that of solvent-rich gel systems.

The ionic conductivity values on the order of 10^–5^ S cm^–1^ are still relevant for thin-film electrochemical devices, electrochromic systems, proof-of-concept solid-state devices, low-current applications, and transparent multifunctional coatings. In addition, for thin electrolyte layers, the effective device resistance can remain acceptable even when the intrinsic conductivity is moderate. Although the ionic conductivity of the present electrolyte is lower than that of highly optimized solvent-rich GPE systems, the developed films demonstrate a favorable balance between electrochemical functionality, optical transparency, and scalable ultrasonic spray processability.

The present study focused on the successful deposition of uniform electrolyte thin films, ionic conductivity behavior, optical transparency, controllable film thickness, and process suitability for future device integration. Thus, this work should be considered a materials/process platform study. Given the moderate ionic conductivity and transparent thin-film nature of the electrolyte, the system is expected to be suitable for low-to-moderate switching speed applications, transparent smart window prototypes, low-power electrochromic devices, and large-area coatings where process scalability is critical. Future plans for device-level validation include switching speed, coloration efficiency, and cycling stability.

## 4 Conclusions

In this experiment, lithium-containing PEG/HPMC polymer electrolyte films were prepared using ultrasonic spray deposition technology. LiClO_4_ and LiCl were added into the polymer as lithium salts, separately. Their transmittance and lithium ionic conductivity correlated to the spray parameters, such as lithium salt concentration, solution flow rate, and spray coats, were investigated. The optimal preparation condition of the LiClO_4_ system was 0.7 M LiClO_4_, a spray flow rate of 0.5 mL min^–1^, and 20 coats. Its 97.5% transmittance and 3.56 × 10^–5^ S cm^–1^ ionic conductivity make it suitable for electrochromic windows. In the LiCl system, the optimal preparation condition was 0.4 M LiCl, a spray flow rate of 0.5 mL min^–1^, and 20 coats. Its 91.1% transmittance and 1.07 × 10^–5^ S cm^–1^ ionic conductivity also make it another candidate material for electrochromic electrolytes. These gel polymer electrolytes with high transmittance and high ionic conductivity were successfully prepared using ultrasonic spray deposition technology, making them capable of electrochromic applications. The optical and electrochemical properties of the electrolytes prepared in this study can be effectively controlled by changing the lithium salt concentration and spray parameters. Compared with electrolytes prepared by traditional methods, the polymer electrolyte films developed in this study exhibit superior performance. The combination of PEG/HPMC gel polymer and ultrasonic spray deposition technology is beneficial for developing electrolytes with promising applications in the electrochromic market.
